# New Findings on the Hosts and Distribution of *Eustrongylides excisus* (Nematoda: Dioctophymatidae) and Other Zoonotic Parasites in Fish Species from an Uninvestigated Subalpine Lake, Varese Lake (Northwestern Italy)

**DOI:** 10.3390/pathogens15050475

**Published:** 2026-04-28

**Authors:** Michele Macrelli, Martina Ossola, Giovanni Sala, Damiano Accurso, Monica Caffara, Andrea Gustinelli, Marco Farioli, Cristian Salogni

**Affiliations:** 1 Istituto Zooprofilattico Sperimentale della Lombardia e dell’Emilia Romagna “Bruno Ubertini”, 25124 Brescia, Italy; michele.macrelli@izsler.it (M.M.); giovanni.sala@izsler.it (G.S.); damiano.accurso@izsler.it (D.A.); cristian.salogni@izsler.it (C.S.); 2 Department of Veterinary Medical Sciences, Alma Mater Studiorum University of Bologna, 40064 Ozzano dell’Emilia, Italy; monica.caffara@unibo.it (M.C.); andrea.gustinelli2@unibo.it (A.G.); 3 Department of Veterinary Public Health and Food Safety of Animal Origin Products, Agenzia di Tutela della Salute dell’Insubria, 21100 Varese, Italy; veterinarivarese@ats-insubria.it

**Keywords:** *Eustrongylides excisus*, fish-borne zoonotic parasites, freshwater fish, Lake Varese, epidemiology

## Abstract

The increasing consumption of fish has raised public health concerns regarding fish-borne zoonotic helminths (FBZHs), which are recognized as significant food-borne parasites worldwide. In freshwater environments, *Clinostomum complanatum*, *Opisthorchis felineus*, *Pseudamphistomum truncatum*, *Dibothriocephalus latus* and *Eustrongylides excisus* are of particular concern in Italy and neighbouring countries. This study aimed to assess the prevalence of these FBZHs in five commercially and ecologically relevant freshwater fish species from Lake Varese, a heavily anthropized and understudied basin in northern Italy. A total of 59 fish were examined via necropsy and stereomicroscopic inspection of skeletal muscles. Only *Eustrongylides* spp. larvae were detected, with a prevalence of 16.9%. Molecular analysis (ITS region) identified them as *E. excisus*. This study reports, for the first time in Western Europe, *E. excisus* in *Sander lucioperca* (*p* = 12.5%) and *Esox lucius* (*p* = 8.3%). The highest prevalence occurred in *Silurus glanis* (*p* = 37.5%), followed by *Perca fluviatilis* (*p* = 25.0%), while *Tinca tinca* showed no infection. These findings confirm that among the FBZHs considered, *E. excisus* is currently present and expanding both in host range and geography in Italian lakes, underscoring the need for updated epidemiological data to support risk assessment, food safety and zoonotic parasite control in freshwater fisheries.

## 1. Introduction

In recent decades, the global demand for fish and seafood has steadily increased. This trend is driven by shifting dietary habits, increased health awareness, and culinary practices that include raw or lightly cooked fish. As a result, fish-borne parasitic infections have become an emerging concern for food safety and public health [1]. According to the Food and Agriculture Organization (FAO), global per capita fish consumption has reached approximately 20.7 kg per year [2], with an upward trend especially evident in Europe. This rising consumption has amplified awareness of the risks posed by fish-borne zoonotic helminths (FBZHs), which are now recognized among the most significant food-borne parasites worldwide. More than 50 species of FBZHs are known to cause human infections through the consumption of raw or undercooked fish containing viable parasites [3]. According to the most recent available estimates, the World Health Organization (WHO) reported around 56 million cases of parasitic infection linked to fish consumption in 2012 [4].

While most attention has historically focused on marine species, helminths in freshwater fish also pose relevant zoonotic risks. All wild-caught freshwater fish should be considered potential reservoirs of viable zoonotic parasites [5]. Although freshwater fisheries represent a small share of total consumption in the EU, they remain highly significant for lake-dwelling populations and tourists seeking traditional lake-based dishes [3].

Among freshwater FBZHs, *Clinostomum complanatum, Contracaecum rudolphii, Opisthorchis felineus, Pseudamphistomum truncatum, Dibothriocephalus latus* and *Eustrongylides excisus*, have emerged as parasites of particular concern in Europe. These species have been reported in more than 32 fish hosts across various water basin—including rivers and lakes—in Italy (Table 1), France, Switzerland and Germany [3,6,7,8].

Their ability to infect fish species of commercial and dietary relevance, combined with their environmental persistence and zoonotic potential, highlight the urgent need for targeted parasitological surveillance in underinvestigated freshwater environments. One such area is Lake Varese, a mid-sized subalpine lake in northern Italy of ecological and recreational relevance. Updated epidemiological data from this basin are essential to support effective food safety management and guide evidence-based decision-making. Therefore, this study aims to (i) investigate the presence of larvae of *Eustrongylides* spp., *C. complanatum*, *C. rudolphii*, *D. latus*, *O. felineus*, and *P. truncatum* in five wild fish species of commercial and ecological relevance from Lake Varese, and (ii) assess the prevalence and anatomical localization of the detected parasites in the infected specimens.

## 2. Materials and Methods

### 2.1. Study Area

This study was conducted at Lake Varese (45°49′ N, 8°44′ E), located in northwestern Italy (Lombardy region). It is a monomictic subalpine lake of glacial origin located at an elevation of 236 m above sea level [10]. This eutrophic lake has a mean depth of 11 m, a maximum depth of 26 m, a surface area of 14.8 km^2^, a volume of 153 million cubic metres (m^3^), and a theoretical renewal time of 1.7–1.9 years [11,12]. The lake receives inflow from two tributaries, the Brabbia Channel and the Tinella Stream, with annual average discharges of 23 × 10^6^ and 10 × 10^6^ m^3^ yr^−1^ respectively, and has a single outflow, the Bardello Stream, with an annual average discharge of 80.4 × 10^6^ m^3^ yr^−1^ [13]. This basin provides valuable habitats for a wide range of flora and fauna, and it contributes to the local economy through recreational and professional fishing, as well as water-related industries.

### 2.2. Fish Collection

This study was conducted from June 2024 to March 2025, with fish sampled at two sites, one located in the southern part of the lake and the other in the northern part.

Five fish species were selected for this study: pike (*Esox lucius italicus*), pike-perch (*Sander lucioperca*), European perch (*Perca fluviatilis*), tench (*Tinca tinca*), and wels catfish (*Silurus glanis*). These species were selected based on their abundance and position in the lake food chain [14] and their recognized commercial and recreational importance [15,16]. All selected fish species are susceptible to hosting larvae or metacercariae of the zoonotic parasites under investigation. As no prior data were available on the presence of these parasites in Lake Varese, no minimum sample size per species was defined.

Fish were obtained from a local professional fisherman, refrigerated at 0–4 ± 1 °C, and transported to the laboratory for analysis.

### 2.3. Morphological and Anatomopathological Examination

Post mortem examinations were carried out within 12 h of fish collection. Each specimen was identified according to the ichthyofauna identification guidelines of the Italian Ministry of the Environment and Protection of Land and Sea [17], then measured for total length (from the tip of the snout to the tip of the tail) and weighed. External examinations were performed to detect large encysted larvae on the skin, gills, fins, and buccal cavity. Skeletal muscles and internal organs were carefully dissected for visual inspection.

### 2.4. Parasitological Identification and Molecular Analysis

To detect encysted plerocercoid, nematode larvae, and metacercariae, skeletal muscles and subcutaneous tissues were transversely sliced into 5 mm thick sections using a scalpel, compressed between glass slides, and examined by transillumination under a stereomicroscope (Nikon Stereoscopic Microscope SMZ745, Nikon Corporation, Tokyo, Japan), following the protocol described by Smagulova et al. [18]. The entire fillet was examined for all species except for *S. glanis*; due to the large body size of this species, only representative portions of the fillet from the anterior and posterior parts of the body were analysed. Acid–pepsin digestion was not performed. Isolated nematode larvae were subsequently identified at the genus level based on morphological features, following the identification keys by Moravec [19]. All detected parasites were counted, their locations recorded, and observed microscopically (Microscope Eclipse E200, Nikon Corporation, Tokyo, Japan) before preservation in 70% ethanol for subsequent molecular analyses of the ITS rDNA following the protocol of Prearo et al. [20].

### 2.5. Statistical Analysis

Prevalence (*p*), mean intensity (MI), and mean abundance (MA) of *Eustrongylides* spp. infestation were calculated following Bush et al. [21]. Prevalence values are presented with their 95% confidence intervals (CIs), calculated according to Bush et al. [21].

To explore potential differences in parasitism between the two sampling locations, Fisher’s exact test was applied, and odds ratios (ORs) with 95% confidence intervals (CI) were computed.

Due to the limited overall sample size and the unbalanced distribution of individuals across fish species—particularly the very low number of infected specimens in several groups—the assumptions required for valid inferential statistical analyses were not met. Therefore, no formal statistical tests were performed, and the results are presented descriptively. The significance level that would have been applied for inferential analyses was set at α = 0.05.

Descriptive statistics, including mean values and standard deviations (SDs) for fish weight and length, were computed for each species. All statistical analyses and data management were performed using Microsoft Excel (version 2019).

## 3. Results

### 3.1. Fish Collection Results

A total of 59 fish were collected: 12 specimens of *E. lucius*, 8 specimens of *S. lucioperca*, 19 specimens of *P. fluviatilis*, 4 specimens of *T. tinca*, and 16 specimens of *S. glanis*.

Slightly more than half of the specimens were collected from the northern part of the lake (52.5%; 31/59). Most specimens (67.8%; 40/59) were captured during the summer months of 2024, while the remaining fish were collected in spring (15.3%; 9/59), autumn (15.3%; 9/59), and winter (1.7%; 1/59) until March 2025. The variation in fish numbers across seasons reflects both practical constraints and ecological factors. For example, heavy rainfall throughout the 2024 fishing season [22] reduced fishing effort and the number of successful catches in winter. The uneven sampling distribution across seasons did not allow for an assessment of seasonal trends.

Differences in the number of individuals collected per species may reflect their relative abundance in the lake, although species-specific seasonal behaviors could also have contributed.

### 3.2. Morphological and Anatomopathological Results

This study included specimens of various ages, resulting in a wide standard deviation (SD) for both weight and total length within each species (Table 2).

At the anatomopathological level, the only observed cutaneous alterations were skin reddening, cutaneous and fin lacerations, all consistent with handling and capture. No lesions suggestive of disease were observed in the gills or internal organs of any specimens analyzed.

### 3.3. Parasitological Identification and Molecular Analysis Results

The only FBZHs observed were reddish *Eustrongylides* spp. nematode larvae, detected in 16.9% of the fish (10/59) (Table 2). The prevalence of *Eustrongylides* spp. was 37.5% in *S. glanis* (95% CI: 18.5–61.4), 10.5% in *P. fluviatilis* (95% CI: 2.9–31.4), 12.5% in *S. lucioperca* (95% CI: 2.2–47.1), and 8.3% in *E. lucius* (95% CI: 1.5–35.4). No larvae were detected in *T. tinca*. The mean intensity of infestation ranged from 1 (*S. lucioperca; E. lucius*) to 6.3 (*S. glanis*), while mean abundance ranged from 0.13 (*S. lucioperca*) to 2.75 (*S. glanis*).

Larvae were located in the coelomic cavity of *E. lucius* and *S. glanis*, and in the muscles of *S. lucioperca* and *P. fluviatilis.* The highest level of infestation in the coelomic cavity was observed in a *S. glanis* hosting 14 larvae, while the greatest number in musculature was found in a *P. fluviatilis* with 4 larvae, all situated in the antero-ventral region. The larvae measured 2.0–5.0 cm and were all detected macroscopically, with no additional larvae found under stereomicroscopic examination.

The ITS region of the rDNA was successfully amplified and sequenced. Sequence similarity searches performed using the NCBI BLAST+ (https://blast.ncbi.nlm.nih.gov/Blast.cgi, accessed on 18 March 2026) against the NCBI nucleotide database showed 100% identity with reference sequences of *E. excisus* available in GenBank, allowing for the identification of the specimens at the species level and confirming the morphological identification of the larvae collected. The obtained ITS rDNA sequences were deposited in GenBank under accession number PZ314426.

Subjects sampled in the northern site showed a higher prevalence of parasitism (25.8%; 8/31) compared to those from the southern site (7.1%; 2/28). Although Fisher’s exact test did not reveal a statistically significant association between geographic area and parasitism, an odds ratio of 4.52 (95% CI: 0.87–23.50; Fisher’s exact test *p* = 0.0838) suggests a trend toward a higher risk of parasitism in subjects from the North.

Due to the limited overall sample size and the low number of infected individuals per species (ranging from 0 to 6), no statistically robust comparison of infection levels among fish species could be performed.

No larvae of *C. complanatum, O. felineus, P. truncatum,* and *D. latus* were detected.

## 4. Discussion

The results of this study provide the first evidence of *E. excisus* in Lake Varese. This parasite was first reported in an Italian subalpine lake in 2020 and then identified in the nearby Lake Maggiore in 2023 [20]. These findings suggest that *E. excisus* may have already been present in Lake Varese but remained undetected due to a lack of targeted investigations. Indeed, recent research has documented an expanding distribution of *E. excisus* in Italy, potentially driven by the rising population of cormorants (*Phalacrocorax carbo sinensis*) around inland water bodies [23]. A marked rise in the breeding population of cormorants has also been documented around Lake Varese since 2002. The increase is mainly attributed to the proximity of the lake to a Marsh Nature Reserve, which serves as a major nesting site for waterbirds [24]. In 2021, more than 500 cormorant nests were recorded in this Reserve during the winter season. This large and largely sedentary cormorant population may contribute to the presence of *E. excisus* in nearby, still underinvestigated water bodies. In response to this population growth and its documented impact on freshwater fish communities, the Lombardy Regional Authority implemented control measures, including targeted culling [25]. Continued monitoring will be essential to assess whether these measures also affect the distribution and prevalence of *E. excisus* in the region.

As described by Castiglione et al. [26], water eutrophication and increasing temperatures are additional risk factors that may have contributed to creating a favourable environment for the survival and expansion of *Eustrongylides* spp. in Italian lakes.

*E. excisus* were isolated from four out of five freshwater fish species selected for their commercial and ecological relevance in this area. This study is the first to report *S. lucioperca* and *E. lucius* as hosts for *E. excisus* in Western Europe. Previously, *E. excisus* was reported in pike-perch in Serbia [27] and Ukraine [28], with prevalence rates of 14.0% (3/21) and 58.1% (43/74), respectively. *Eustrongylides* spp. were reported in the European pike in Ukraine [28], with a prevalence rate of 58.9% (69/117), considerably higher than the 8.3% recorded in our study.

*S. glanis* showed the highest prevalence of *E. excisus* infection (37.5%) and the highest mean intensity (6.3). Similar findings were reported in Lake Massaciuccoli in central Italy (*p* = 75.0%; 42/56; MI = 5.7) [26].

No comparable data are available for *S. glanis* in other subalpine lakes in northern Italy. Given its feeding habits, which include fish and invertebrates, and its capacity to rapidly adapt to new habitats, *S. glanis* may represent an ideal target species for assessing the presence of *Eustrongylides* spp. in the freshwater basins investigated.

The prevalence observed in *P. fluviatilis* (10.5%) was comparable to that reported for the same species in other subalpine lakes in northern Italy, ranging from 4.3% (3/70) to 25.3% (38/150) [23]. The detection of *Eustrongylides* spp. larvae in the musculature of *P. fluviatilis* is particularly significant, as this species is often consumed raw (e.g., perch carpaccio).

Analysis indicated that juvenile individuals were also infected. Due to their small size and susceptibility to predation by definitive hosts, these individuals play an important role in perpetuating the life cycle of *Eustrongylides* spp. In contrast, larger fish, such as wels catfish, are generally too large to be preyed upon by piscivorous birds and may therefore act as a dead end for parasite transmission. Large predatory fish like *S. glanis*, serving as paratenic hosts, can concentrate the parasitic load.

No *Eustrongylides* spp. larvae were found in *T. tinca*; however, due to the limited number of tench specimens examined, the presence of this parasite in this species cannot be ruled out. To date, only one study in central Italy reported tench as a potential host for *Eustrongylides* spp., with a very low prevalence (0.13%; 6/19,809) [29]. This suggests that tench (family Tincidae) may be among the less susceptible species, as they mainly feed on plant material and zooplankton [30]. Accordingly, tench could be considered an accidental host, acquiring infection only in heavily contaminated environments [31].

As highlighted in the present study, a comprehensive visual examination of both the coelomic cavity and skeletal muscles represents an important and practical method for detecting *Eustrongylides* spp. larvae in infected fish. Although the musculature is frequently reported as the primary site for larval detection, larvae may also be present in other anatomical locations, as observed in *S. glanis* in this study. In this species, 3–5 cm reddish were identified in the coelomic cavity, and in some cases could be mistaken for blood vessels. Interestingly, a similar larval distribution was reported in *S. glanis* from Lake Massaciuccoli [26], suggesting that *Eustrongylides* spp. Larvae may have difficulty migrating from the coelomic cavity to their predilection site in this host.

According to European legislation, the presence of visible parasites during visual inspection renders fish unfit for human consumption [32]. Clinical symptoms of human eustrolngyliasis typically appear within 24 h after ingestion of raw or undercooked freshwater fish harbouring third- or fourth-stage larvae [33]. The disease usually manifests with gastrointestinal symptoms, including acute abdominal pain and gastritis, although rare cases may progress to intestinal perforation. Extraintestinal signs, including subcutaneous or cutaneous nodules, have also been reported [23]. Although limited data are available on the impact of different processing methods on the survival of *Eustrongylides* spp. larvae in freshwater fish, cooking at 60 °C at the core for 10 min or freezing at –20 °C at the core for 24 h appears to be effective in inactivating the parasite [26].

With the exception of *D. latus,* this is the first study to investigate the presence of *C. complanatum*, *O. felineus*, *P. truncatum*, and *C. rudolphii*, in Lake Varese. *D. latus* was first identified in *P. fluviatilis* in 1955 (*p* = 80%) and later in 1973 (*p* = 13.5%) [7]. In 1967, a water restoration project was initiated by the Varese Province to reduce external nutrient loads. Measures included the construction of a sewage collection network, an O-ring diversion system, and a centralized wastewater treatment plant. Wastewaters received tertiary treatment in 1986, and water diversion was completed in 1994 [34].

The absence of *D. latus* plerocercoid larvae in fish from Lake Varese was previously reported in 2001 [35] and 2009 [36], and our data confirm this result. These findings contrast with the epidemiological data obtained from other large subalpine lakes near Lake Varese, such as Lake Maggiore, Como, and Iseo, where *D. latus* is considered endemic [7]. The spatial distribution of *D. latus* in these lakes has been linked to ineffective sewage treatment systems, which allow for contamination by eggs released from infected humans [37].

The absence of *O. felineus* metacercariae in the fish analysed was expected. In Italy, *O. felineus* metacercariae have been reported only in the muscles *T. tinca* from Lakes Bolsena and Bracciano, with prevalences of 75% (33/44) and 95% (83/87) respectively. Between 2003 and 2011, 211 human cases of opisthorchiasis were linked to the consumption of raw tench fillets from these lakes. Epidemiological investigations following these outbreaks identified snails of the genus *Bithynia* and *T. tinca* as intermediate hosts, and stray cats along the lake shores as definitive hosts. The stray cat population has been estimated at 280–360 individuals around Lake Bracciano and approximately 1000 around Lake Bolsena [38]. Lake Varese, with a lower number of tench and fewer stray cats along its shores, can therefore be considered at lower risk for *O. felineus*, a conclusion supported by our data.

Humans can play a significant role in the epidemiology of this parasite. In particular, practices such as releasing small tench on lakeshores or improperly disposing of fish leftovers from restaurants can facilitate the parasite’s life cycle by allowing infected fish to be consumed by cats and dogs, which serve as definitive hosts [6].

*P. truncatum* and *C. complanatum* have recently been reported in Italy, respectively in *T. tinca* and *P. fluviatilis*, in two other subalpine lakes of the Lombardy region that share ecological characteristics with Lake Varese, including comparable fish communities, similar intermediate hosts and analogous shoreline vegetation [39,40]. However, neither *P. truncatum* nor C*. complanatum* were detected in the fish analysed in the present study. Further investigations are needed to identify the biological and ecological factors influencing the geographical expansion of these food-borne trematodes.

## 5. Conclusions

The present study highlights the importance of monitoring underinvestigated freshwater ecosystems, which may serve as reservoirs for zoonotic parasites. This study reports, for the first time, the presence of *E. excisus* in pike-perch (*S. lucioperca*) and northern pike (*E. lucius*) in Western Europe, confirming its ongoing geographic and host expansion across Italian lakes. These findings underline the growing public health relevance of this fish-borne zoonotic parasite.

Although no human cases of eustrongyliasis have been documented in Europe to date, veterinary authorities must continue to raise awareness and provide training to relevant stakeholders to prevent the emergence of this and other freshwater food-borne parasitic diseases. A comprehensive visual examination of both the coelomic cavity and skeletal muscles remains an effective and practical approach for detecting *Eustrongylides* spp. larvae and should be systematically performed during inspections of fish from Italian lakes.

Early detection and surveillance in underinvestigated freshwater ecosystems are crucial for identifying reservoirs of FBZHs and developing ad hoc control strategies to prevent disease outbreaks and limit their spread. Controlling these parasites requires a One Health approach, based on strong collaboration between the human, animal and environment health sectors. Enhancing water quality and wastewater infrastructure, managing interactions between intermediate and definitive hosts, and educating the public to interrupt parasite transmission cycles are key strategies for their control.

## Figures and Tables

**Table 1:** Life cycle, host range and human cases in Italy of the investigated parasites.

Parasite	Class	1st Intermediate Host	2nd Intermediate Host	Final Host	Human Susceptibility	Human Cases in Italy
*Eustrongylides* spp.	Nematode	Aquatic oligochaetes	Planktivorous, benthivorous freshwater fish	Piscivorous birds	Accidental host	None reported
*D. latus*	Cestode	Crustacean copepods	Freshwater fish	Fish-eating mammals	Can act as definitive host	Since 1980: 2–10 cases/year (Subalpine lakes) [7]
*O. felineus*	Trematode	Freshwater gastropods (Genus *Bithynia*)	Freshwater fish (Cyprinid)	Fish-eating mammals	Can act as definitive host	Since 2003: periodic outbreaks (Central Italy) [9]
*P. truncatum*	Trematode	Freshwater gastropods (Genus *Bithynia*)	Freshwater fish (Cyprinid)	Fish-eating mammals	Can act as definitive host	None reported
*C. complanatum*	Trematode	Freshwater gastropods (Lymnaeidae)	Freshwater fish, amphibians	Fish-eating mammals and birds	Accidental host	None reported

**Table 2:** Total length (cm) and weight (g) of fish collected (No.) Prevalence (*p*), mean intensity (MI) and mean abundance (MA) of *Eustrongylides* spp. infestation, their standard deviations (SDs), and location of *Eustrongylides* spp.

Fish Species	Fish Specimen Data					*Eustrongylides* spp.			
	Collected No.	Weight (g)	Weight SD (g)	Length (cm)	Length SD (cm)	*p*	MI	MA	Location
*T. tinca*	4	1768.5 ± 1068.9	1090.7	41.8 ± 15.0	15.4	0 (0/4)	-	-	-
*E. lucius*	12	1575.1 ± 727.9	1286.5	57.2 ± 10.9	19.2	8.3% (1/12)	1	0.08	coelomic cavity
*S. lucioperca*	8	328.5 ± 250.3	361.2	30.5 ± 6.3	9.1	12.5% (1/8)	1.0	0.13	muscles
*P. fluviatilis*	19	70.0 ± 7.3	16.3	17.4 ± 0.5	1.1	10.5% (2/19)	2.5	0.26	muscles
*S. glanis*	16	4307.6 ± 1393.6	2844.1	81.6 ± 12.9	26.3	37.5% (6/16)	6.3	2.75	coelomic cavity
Total	59					16.9% (10/59)			

## Data Availability

The data presented in this study are available on request from the corresponding author due to institutional data protection policies.

## Abbreviations

The following abbreviations are used in this manuscript:

CI	Confidence Interval
FAO	Food and Agriculture Organization
FBZH	Fish-borne zoonotic helminth
ITS	Internal Transcribed Spacer
MA	Mean abundance
MI	Mean intensity
No.	Number
P	Prevalence
SD	Standard deviation
WHO	World Health Organization
